# Propagation of beta/gamma rhythms in the cortico-basal ganglia circuits of the parkinsonian rat

**DOI:** 10.1152/jn.00629.2017

**Published:** 2018-01-10

**Authors:** Timothy O. West, Luc Berthouze, David M. Halliday, Vladimir Litvak, Andrew Sharott, Peter J. Magill, Simon F. Farmer

**Affiliations:** ^1^Centre for Mathematics and Physics in the Life Sciences and Experimental Biology (CoMPLEX), Department of Physics and Astronomy, University College London, London, United Kingdom; ^2^Wellcome Trust Centre for Neuroimaging, Institute of Neurology, University College London, London, United Kingdom; ^3^Centre for Computational Neuroscience and Robotics, University of Sussex, Falmer, United Kingdom; ^4^UCL Great Ormond Street Institute of Child Health, London, United Kingdom; ^5^Department of Electronic Engineering, University of York, York, United Kingdom; ^6^Medical Research Council Brain Network Dynamics Unit, University of Oxford, Oxford, United Kingdom; ^7^Oxford Parkinson’s Disease Centre, University of Oxford, Oxford, United Kingdom; ^8^Department of Neurology, National Hospital for Neurology & Neurosurgery, London, United Kingdom; ^9^Sobell Department of Motor Neuroscience and Movement Disorders, Institute of Neurology, University College London, London, United Kingdom

**Keywords:** basal ganglia, connectivity, local field potential, Parkinson’s disease, synchronization

## Abstract

Much of the motor impairment associated with Parkinson’s disease is thought to arise from pathological activity in the networks formed by the basal ganglia (BG) and motor cortex. To evaluate several hypotheses proposed to explain the emergence of pathological oscillations in parkinsonism, we investigated changes to the directed connectivity in BG networks following dopamine depletion. We recorded local field potentials (LFPs) in the cortex and basal ganglia of rats rendered parkinsonian by injection of 6-hydroxydopamine (6-OHDA) and in dopamine-intact controls. We performed systematic analyses of the networks using a novel tool for estimation of directed interactions (nonparametric directionality, NPD). We used a “conditioned” version of the NPD analysis that reveals the dependence of the correlation between two signals on a third reference signal. We find evidence of the dopamine dependency of both low-beta (14–20 Hz) and high-beta/low-gamma (20–40 Hz) directed network interactions. Notably, 6-OHDA lesions were associated with enhancement of the cortical “hyperdirect” connection to the subthalamic nucleus (STN) and its feedback to the cortex and striatum. We find that pathological beta synchronization resulting from 6-OHDA lesioning is widely distributed across the network and cannot be located to any individual structure. Furthermore, we provide evidence that high-beta/gamma oscillations propagate through the striatum in a pathway that is independent of STN. Rhythms at high beta/gamma show susceptibility to conditioning that indicates a hierarchical organization compared with those at low beta. These results further inform our understanding of the substrates for pathological rhythms in salient brain networks in parkinsonism.

**NEW & NOTEWORTHY** We present a novel analysis of electrophysiological recordings in the cortico-basal ganglia network with the aim of evaluating several hypotheses concerning the origins of abnormal brain rhythms associated with Parkinson’s disease. We present evidence for changes in the directed connections within the network following chronic dopamine depletion in rodents. These findings speak to the plausibility of a “short-circuiting” of the network that gives rise to the conditions from which pathological synchronization may arise.

## INTRODUCTION

**Fig. 1.**
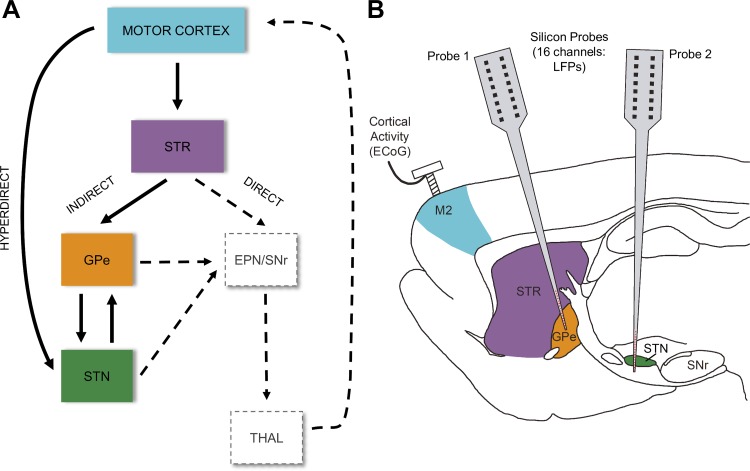
Cortical-basal ganglia circuits and experimental paradigm. *A*: schematic of canonical cortical-basal ganglia circuit incorporating the antagonistic direct and indirect pathways first described by Albin et al. (1989), as well as the cortico-subthalamic hyperdirect pathway (Nambu et al. 2002). The motor cortex (M2; blue) has major inputs to the basal ganglia (BG) at the striatum (STR; purple) and subthalamic nucleus (STN; green). Information flow along the indirect pathway is routed via the external segment of the globus pallidus (GPe; orange). The indirect, direct, and hyperdirect pathways ultimately impinge on the output nuclei of the BG, made up of the entopeduncular nucleus (EPN) and substantia nigra pars reticulata (SNr). BG output targets thalamic relays, some of which return back to motor cortex. Brain structures from which neuronal signals were recorded in this study are delineated by solid boxes, with solid arrows indicating the connections (interactions) that were analyzed. Other structures and interactions are delineated by dashed boxes and arrows, respectively. *B*: diagram of the recording configuration in anesthetized rats. Local field potentials (LFPs) were recorded from the BG using 2 multichannel silicon probes; the first probe was targeted to the STR and GPe, whereas the second probe was targeted to STN. Electrocorticograms (ECoG) were recorded with a screw positioned over the “secondary motor cortex” (M2). Boundaries and positioning are approximate.

The basal ganglia (BG) are host to a small but important cluster of dopaminergic neurons that act to modulate the activity of a large reentrant network that comprises the cortico-basal ganglia-thalamo-cortical circuit (DeLong and Wichmann 2010; Lanciego et al. 2012). Investigation of the structure of this network (Bolam et al. 2000; Smith et al. 1998 has led to what has become a canonical view of the circuit (depicted in Fig. 1*A*) and has formed the basis from which a number of process theories of BG function have arisen (for a review, see Schroll and Hamker 2013).

Recent theory concerning the organization of brain networks and communication within them via synchronized oscillations (Bressler and Menon 2010; Fries 2005, 2015; Thut et al. 2012; Varela et al. 2001) has emphasized the importance of understanding the dynamics of these networks beyond that afforded by studying structural connectivity alone (Deco et al. 2008, 2012). Neural oscillations and their synchronization have been measured across multiple spatial scales of brain activity, from single neuronal discharges up to the level of mesoscale neural ensembles such as those measured in the local field potential (LFP) or electrocorticogram (ECoG). Moreover, dysregulations of oscillations and inter-areal synchrony have been reported in brain disorders such as Parkinson’s disease (PD), schizophrenia, and epilepsy, leading to the hypothesis that the oscillations themselves bear a causal role in the behavioral impairments associated with these pathologies (Hammond et al. 2007; Schnitzler and Gross 2005; Uhlhaas and Singer 2006).

Excessive beta oscillations (14–30 Hz) in the BG associated with dopamine depletion have been observed reliably in untreated patients with PD (Brown et al. 2001; Hammond et al. 2007; Levy et al. 2000; Weinberger et al. 2006). Beta rhythms are attenuated by treatments such as dopamine replacement therapy (Beudel et al. 2017; Kühn et al. 2006; Levy et al. 2002; Weinberger et al. 2006; West et al. 2016) and deep brain stimulation (DBS) (Eusebio et al. 2011; Ray et al. 2008; Whitmer et al. 2012) in a way that correlates with the degree of improvement of akinetic/rigid motor symptoms. This has strengthened the argument that the pathological beta rhythms are directly related to the functional impairment seen in patients (Brittain and Brown 2014; Hanslmayr et al. 2012). Furthermore, gamma activity in the motor system has been hypothesized to be prokinetic (Schoffelen et al. 2005). In PD, the spectral power of multiunit recordings from the subthalamic nucleus (STN) at 40–90 Hz have been demonstrated to be negatively correlated with bradykinetic symptoms in patients (Sharott et al. 2014).

The pathological oscillations observed in mesoscale electrophysiological signals are a direct consequence of changes to the underlying networks of neuronal ensembles that generate them. This understanding has led to the reclassification of multiple neurological diseases such as PD or Tourette’s as “circuit disorders” (DeLong and Wichmann 2010). Knowledge of how dopamine depletion results in changes to the network, and the subsequent emergence of pathological synchrony, is likely to lead to a better understanding of the causes of impairment and its treatments (Schroll et al. 2014; Shen et al. 2008). Thus improving insight into how changes in network organization lead to the emergence of pathological dynamics is an important line of enquiry (Dostrovsky and Bergman 2004; Holgado et al. 2010; Wichmann and DeLong 1999).

Previous work aiming to understand the origins of the pathological beta rhythm has involved systematic lesioning of the BG network (Ni et al. 2000; Tachibana et al. 2011), computational modeling (Holgado et al. 2010; Lienard et al. 2017; Marreiros et al. 2013; Moran et al. 2011; Nevado-Holgado et al. 2014; Pavlides et al. 2015), and techniques from signal analysis (Litvak et al. 2011; Mallet et al. 2008a, 2008b; Sharott et al. 2005a). In this article, we take the latter approach and, through analysis of neural recordings, aim to infer the changes in neural transmission that occur in cortico-BG circuits following chronic dopamine depletion.

Connectivity between parts of the brain can be inferred from the statistical dependencies that arise due to neural transmission; we refer to this as “functional connectivity” as per Friston (2011). Previous studies have aimed to describe “effective” connectivity (i.e., causal interactions) within this network and have employed the dynamic causal modeling (DCM) framework to do so. To date, two such studies have utilized the inversion of biophysical models upon cross-spectral densities from recordings in either anesthetized 6-hydroxydopamine (6-OHDA)-lesioned rats (Moran et al. 2011) or awake DBS patients (Marreiros et al. 2013). Both found evidence for the strengthening of the cortico-subthalamic connection (termed the “hyperdirect” pathway (Nambu et al. 2002) in the dopamine-depleted state.

From this work, among others, several hypotheses have arisen concerning the emergence of pathological beta rhythms as a result of the dopamine depletion associated with PD (for a review see Holgado et al. 2010). These include the dopamine-dependent modulation of recurrent loops within the network, either between the reciprocally coupled network of neurons of the STN and the external globus pallidus (GPe) (Bevan et al. 2002; Holgado et al. 2010; Liu et al. 2017; Plenz and Kital 1999; Terman et al. 2002) or of a longer loop involving feedback from BG output nuclei to the cortex via thalamocortical tracts (Leblois et al. 2006; Pavlides et al. 2012, 2015). Alternatively, it has been proposed that dopamine depletion disrupts mechanisms that regulate the gain of cortical afferents to the BG and somehow disrupt striatal outflow (Brown 2007; Hammond et al. 2007). The striatum (STR) itself has also been implicated in the generation of pathological beta rhythms, either through alterations to its internal dynamics (Damodaran et al. 2015; McCarthy et al. 2011) or via increased striatal inhibition of targets in the GPe that act to promote beta synchrony (Gillies and Willshaw 2004; Kumar et al. 2011).

Using a recently described nonparametric (model free) signal analysis technique (Halliday 2015), we studied the effects of dopamine depletion on neural connectivity in the network formed by elements of the BG and motor cortex in 6-OHDA-lesioned and dopamine-intact control rats. We employed this method as a measure of directed functional connectivity (hereafter shortened to “directed connectivity”). It is a model-free estimate that makes no assumptions as to the causes of the data (for discussion see Bastos and Schoffelen 2016), only that temporal precedence implies a driving neuronal influence (please see *Limits to inference of causal interactions and mechanisms from neurophysiological signals alone* for discussion). Furthermore, we use a multivariate extension of the framework (Halliday et al. 2016) to determine whether the interaction between two areas shares correlation with activity recorded at a third structure in the network. This approach provides insight into frequency-specific directional connectivity and the degree to which transmission between two coupled regions are autonomous of another reference region. By recording LFPs and ECoG in 6-OHDA-lesioned animals and dopamine-intact controls, we aim to identify changes to connectivity that occur as a result of the loss of dopamine from these circuits. Our findings are interpreted within the context of the canonical circuit (Fig. 1*A*), as well as other existing models of basal ganglia connectivity, and several hypotheses concerning the generation and propagation neural rhythms in the network.

## METHODS

### Experimental Data

#### Electrophysiological recordings.

Experimental procedures were carried out on adult male Sprague-Dawley rats (Charles River, Margate, UK) and were conducted in accordance with the Animals (Scientific Procedures) Act, 1986 (UK). Recordings were made in eight dopamine-intact control rats (288–412 g) and nine 6-OHDA-lesioned rats (285–428 g at the time of recording), as described previously (Magill et al. 2006; Mallet et al. 2008a, 2008b; Moran et al. 2011). Briefly, anesthesia was induced with 4% (vol/vol) isoflurane (Isoflo; Schering-Plough, Welwyn Garden City, UK) in O_2_ and maintained with urethane (1.3 g/kg ip; ethyl carbamate; Sigma, Poole, UK) and supplemental doses of ketamine (30 mg/kg; Ketaset; Willows Francis, Crawley, UK) and xylazine (3 mg/kg; Rompun; Bayer, Leverkusen, Germany).

The ECoG was recorded via a 1-mm-diameter steel screw juxtaposed to the dura mater above the right frontal cortex [centered at 4.5 mm anterior and 2.0 mm lateral of Bregma, corresponding to the “secondary motor cortex” (M2) of Paxinos and Watson (2007) or the medial agranular field of the somatic sensorimotor cortex of Donoghue and Wise (1982); see Fig. 1*B*] and was referenced against another screw implanted in the skull above the ipsilateral cerebellar hemisphere. Raw ECoG was bandpass filtered (0.3–15,00 Hz, −3 dB limits) and amplified (2,000×; DPA-2FS filter/amplifier; Scientifica, Harpenden, UK) before acquisition (see below). Extracellular recordings of LFPs in the dorsal striatum (STR), GPe, and STN were simultaneously made in each animal using silicon probes (NeuroNexus Technologies, Ann Arbor, MI); a first probe captured LFPs in STR and GPe, whereas a second probe captured LFPs in the STN (Fig. 1*B*). Each probe had one vertical array of 16 recording contacts (impedance 0.9–1.3 MΩ measured at 1,000 Hz; area ~400 μm^2^), and each contact on a given probe was separated by 100 µm. Recording sites in the BG were verified by post hoc histology, as described previously (Magill et al. 2006; Mallet et al. 2008a, 2008b), as well as by comparisons of recorded unit activity with the characteristic discharges of STR, GPe, and STN neurons in anesthetized dopamine-intact rats and 6-OHDA-lesioned rats (Abdi et al. 2015; Magill et al. 2006; Mallet et al. 2008a, 2008b; Sharott et al. 2017).The same two probes were used throughout these experiments but were cleaned after each experiment in a proteolytic enzyme solution to ensure that contact impedances and recording performance were not altered by probe use and reuse (Magill et al. 2006; Sharott et al. 2017). Monopolar probe signals were recorded using high-impedance unity-gain operational amplifiers (Advanced LinCMOS; Texas Instruments, Dallas, TX) and were referenced against a screw implanted above the contralateral cerebellar hemisphere. After initial amplification, extracellular signals were further amplified (1,000×) and low-pass filtered at 6,000 Hz using programmable differential amplifiers (Lynx-8; Neuralynx, Tucson, AZ). The ECoG and probe signals were each sampled at 17.9 kHz using a single Power1401 analog-to-digital converter (with integrated ADC16 expansion units) and a personal computer running Spike2 acquisition and analysis software (Cambridge Electronic Design, Cambridge, UK). All signals recorded in a given experimental epoch were captured in a single data file. This, together with the use of a fixed/consistent sampling rate and a single acquisition interface, ensured accurate synchronization (temporal alignment) of cortical and BG signals.

**Fig. 2.**
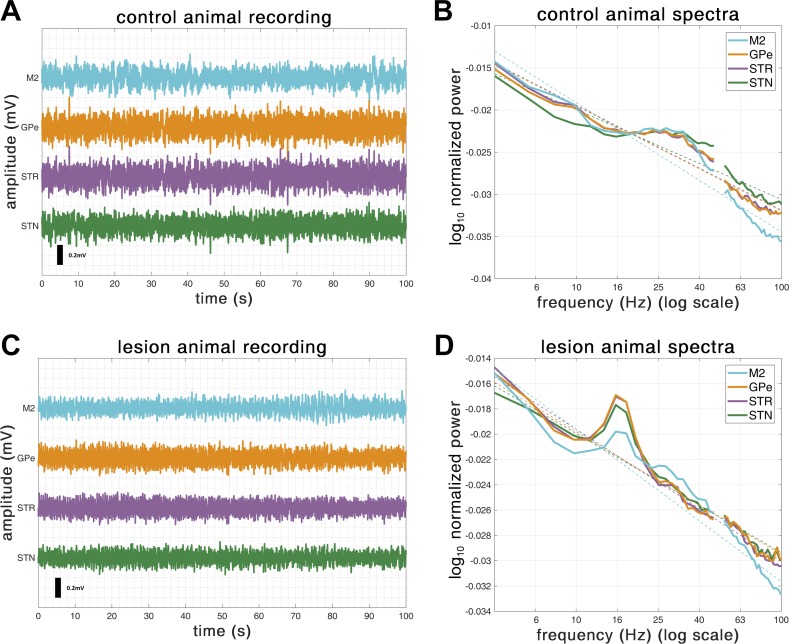
Example recordings of subcortical monopolar LFP and cortical ECoG signals for a single animal from either the control (*A* and *B*) or the 6-OHDA-lesioned (*C* and *D*) groups. *A*: a 100-s sample of LFPs recorded from one dopamine-intact control animal. The example trace shows the time course of LFPs recorded using silicon electrodes implanted in the external globus pallidus (GPe; orange), striatum (STR; purple), and subthalamic nucleus (STN; green). Additionally, electrocorticograms (ECoG) were recorded from a screw positioned over motor cortex (M2; blue). Only raw data are shown. The data were demeaned and then high-pass filtered at 4 Hz. *B*: spectral analysis of example control animal’s recording. Data were epoched into 1-s-long segments; those contaminated by muscle artifact or high-amplitude transients were removed using *Z*-thresholding as described in the text. These epochs were used to construct individual fast Fourier transforms and subsequent periodograms. *C*: same as *A* but for an example 6-OHDA, dopamine depleted animal. The dashed line shows a regression to estimate the 1/*f* background noise. *D*: same as *B* but for a 6-OHDA-lesioned animal.

Neuronal activity was recorded during episodes of spontaneous “cortical activation,” which contain patterns of activity that are similar to those observed during the awake, behaving state (Steriade 2000). Cortical activation was defined according to ECoG activity. Neuronal activity patterns present under this anesthetic regime may only be qualitatively similar to those present in the unanesthetized brain. However, the urethane-anesthetized animal still serves as a useful model for assessing ensemble dynamics within the basal ganglia. Indeed, in 6-OHDA-lesioned animals, exaggerated beta oscillations emerge in cortico-basal ganglia circuits during activated brain states, thus accurately mimicking the oscillatory activity recorded in awake, nonmedicated PD patients. Examples of the raw electrophysiological signals as well the corresponding power spectra for control and lesioned animals are shown in Fig. 2.

#### 6-OHDA lesions of dopamine neurons.

Unilateral 6-OHDA lesions were carried out on 200- to 250-g rats, as described previously (Mallet et al. 2008a, 2008b). Twenty-five minutes before the injection of 6-OHDA, all animals received a bolus of desipramine (25 mg/kg ip; Sigma) to minimize the uptake of 6-OHDA by noradrenergic neurons (Schwarting and Huston 1996a). Anesthesia was induced and maintained with 4% (vol/vol) isoflurane (see above). The neurotoxin 6-OHDA (hydrochloride salt; Sigma) was dissolved immediately before use in ice-cold 0.9% (wt/vol) NaCl solution containing 0.02% (wt/vol) ascorbate to a final concentration of 4 mg/ml. Three milliliters of 6-OHDA solution were then injected into the region adjacent to the medial substantia nigra (4.5 mm posterior and 1.2 mm lateral of Bregma, and 7.9 mm ventral to the dura). The extent of the dopamine lesion was assessed 14–16 days after 6-OHDA injection by challenge with apomorphine (0.05 mg/kg sc; Sigma) (Schwarting and Huston 1996b). The lesion was considered successful in those animals that made >80 net contraversive rotations in 20 min. Electrophysiological recordings were carried out ipsilateral to 6-OHDA lesions in anesthetized rats 21–42 days after surgery, when pathophysiological changes in the basal ganglia are likely to have leveled out near their maxima (Mallet et al. 2008a).

### Data Acquisition and Analysis

#### Data conversion and preprocessing.

To isolate LFPs and ECoGs, all electrophysiological data were downsampled from a hardware native rate of 17.9 kHz to 250 Hz using Spike2 acquisition and analysis software (version 4; Cambridge Electronic Design). Data were then imported from Spike2 into MATLAB (The MathWorks, Natick, MA), where they were analyzed using custom scripts utilizing routines from the FieldTrip software package (contained within SPM 12.3; Oostenveld et al. 2011; http://www.fieldtriptoolbox.org/), as well as Neurospec (http://www.neurospec.org/). Data were preprocessed as follows: *1*) data were first truncated to remove 1 s from either end of the recording; *2*) data were mean subtracted; *3*) data were bandpass filtered with a finite impulse response, two-pass (zero lag) filter designed such that the filter order is rounded to the number of samples for 3 periods of the lowest frequency, between 4 and 100 Hz; *4*) data were then split into 1-s epochs; and *5*) each epoch was subjected to a *Z*-score threshold criterion such that epochs containing any high-amplitude artifacts were removed. Examples of outcomes from this preprocessing are shown in Fig. 2. All ECoG/LFP time series were 90–100 s in duration.

#### Analyses of neurophysiological signals.

##### estimates of spectral power.

Power analyses were made using the averaged periodogram method across 1-s epochs and using a Hanning taper to reduce the effects of spectral leakage. Frequencies between 49 and 51 Hz were removed so that there was no contribution from 50-Hz line noise. The sampling rate of 250 Hz gives a Nyquist frequency of 125 Hz, and 1-s epochs yield Fourier spectra with a 1-Hz frequency resolution and a periodogram resulting from an average of ~100 spectra per channel. All analyses were made using the Neurospec toolbox. Individual spectra were normalized for group-level comparisons by dividing by the total power in the range 4–48 Hz.

##### non-zero phase lag functional connectivity analysis: imaginary coherence.

The commonly used spectral coherence (Halliday et al. 1995) is sensitive to spurious correlations resulting from instantaneous volume conduction between the two signals of interest (Bastos and Schoffelen 2016). This issue is of the most concern when recordings are made in close spatial proximity, such as those between adjacent contacts on the silicon probes used in these experiments. To circumvent this issue, several methods have been developed, such as taking the imaginary part of coherence (Nolte et al. 2004), the phase lag index (PLI) (Stam et al. 2007), or the weighted phase lag index (Vinck et al. 2011). For this study, we initially used the simplest method, the imaginary coherence (iCOH) that is derived from the complex coherency. The more often used coherence is the magnitude-squared coherency. Coherence is real valued on a scale between 0 and 1, with 1 indicating maximal correlation between two signals and 0 indicating an absence of correlation (Halliday et al. 1995). Nolte and colleagues (Nolte et al. 2004) have suggested that by taking the imaginary part of the coherency, the contribution of correlations with zero phase lag (that is, having only a real component) can be negated. This property is shared with the nonparametric directionality (NPD) analysis that we introduce below for estimates of directed connectivity. We note the concerns in Stam et al. (2007) on the validity of imaginary coherence analysis and so include additional analyses based on NPD and use the iCOH metric as a first-pass demonstration that non-zero phase lag interactions are present in the data.

##### nonparametric directionality.

Estimates of directed connectivity were computed using NPD, which is a novel framework to decompose classical, nonparametric Fourier-based coherence estimates by direction (Halliday 2015). Coherence between two random processes, or random signals, is defined as the ratio of the magnitude-squared cross-spectrum between the two signals to the product of their autospectra. It is difficult to infer any directionality from this ratio involving cross-spectra and autospectra. The approach introduced in Halliday (2015) uses optimal minimum mean square error (MMSE) prewhitening of the two signals such that the coherence is calculated from the cross-spectrum only, as the denominator becomes equal to 1. Prewhitening refers to the process of filtering a signal before spectral analysis to make its frequency content closer to white noise.

The prewhitening step generates two new random processes that have spectra equal to 1 at all frequencies and that have the same coherence as the two original signals. The coherence between the prewhitened signals is calculated only from the cross-spectrum between the prewhitened processes, and this is identical to the original coherence. From this MMSE prewhitened cross-spectrum, an inverse Fourier transform generates a time domain correlation measure. This is analogous to the approach used to generate a standard cross-covariance estimate in the time domain, except the MMSE prewhitened time domain correlation measure only has features that occur as a result of the correlation between the signals, effectively removing the confounding influence of the original signals’ autocorrelation.

Three quantities are extracted from this time domain correlation measure according to time lag. These are components with negative time lags, the value at zero time lag, and components at positive time lags. These are used to calculate the strength of correlation in the reverse, zero-lag, and forward directions, respectively. Three inverse Fourier transforms, using the sections over these three lag ranges, are used to obtain the reverse, zero-lag, and forward components of the original coherence estimate. These provide a summative decomposition of the original nonparametric coherence at each frequency into reverse, zero-lag, and forward components.

In this study the zero-lag component is assumed to reflect volume conduction. The forward and reverse components of coherence are used to infer directionality between the different regions. For example, STN activity lagging M2 activity results in a significant forward component of coherence between M2 and STN (with M2 as reference), whereas STN activity leading M2 activity results in a significant reverse component of coherence.

The concept of partial coherence is well established (Medkour et al. 2009; Rosenberg et al. 1998), where coherence is conditioned on a third signal. This conditioning takes the form of a simple linear regression in the frequency domain of each of the two original signals on the third signal or predictor. The resulting partial coherence estimates can be used to test the hypothesis that the pairwise correlation between the original signals can be accounted for by the third signal. The NPD framework is extended to decompose partial coherence into directional components in Halliday et al. (2016). The analysis decomposes the partial coherence into the same three directional components: forward, reverse, and zero lag. The approach is similar to the bivariate case, except MMSE prewhitening is applied using partial autospectra and the partial cross-spectrum.

This analysis can indicate if the signals reflected in the correlation are common to other parts of the network. For example, the partial correlation between A and B with C as predictor can be used to determine if the flow of information A → B is independent of area C or whether the flow of information is A → C → B, in which case the partial coherence between A and B with C as predictor should be zero. The partial coherence also can be used to investigate if the flow of information is C → A and C → B, or if it is A → B → C or C → A → B; in the latter case, the partial coherence and any directional components should be zero.

This assumes that the conditioning signal, C, is representative of the activity in the relevant brain area. If the signal, C, only captures part of the activity in the brain area, then the partial coherence estimate may still have residual features. The most robust interpretation of the partial coherence and multivariate NPD is where the partial coherence (and any directional components) are not significant compared with the directional components for the ordinary coherence. It must be noted that these methods are useful in detecting the linear coupling (additive mixing/linear correlation) of signals. NPD is not suited for detection of nonlinear interactions between signals such as cross-frequency coupling, for instance.

### Statistics and Visualization

To make statistical comparisons of power, connectivity, and directionality spectra between lesioned and control recordings, we used cluster-based permutation testing (Maris and Oostenveld 2007), which avoids introduction of bias through the prior specification of frequency bands. Briefly, the method computes multiple independent *t*-statistics for each sample (channel-frequency pair) between the two experimental conditions (lesion and control). We assume that in regions of the spectra where there is a true physiological difference in the distributions of a metric of interest (i.e., power, iCOH, NPD), there will be a high value of the *t*-statistic in several adjacent frequency bins, and this group of neighboring bins is called a “cluster.”

The purpose of the cluster-based permutation test is to find clusters that are “heavier” (i.e., have a greater sum of *t*-statistic values in the cluster) than could be expected under the null hypothesis. Candidate clusters to be tested are identified by setting a threshold on the *t*-statistic. Importantly, this cluster-forming threshold does not affect the false alarm rate of the test, only the sensitivity to large clusters with smaller *t*-values as opposed to small clusters with large *t*-values. The statistical significance of candidate clusters is then tested by approximating the reference distribution using a large number of permutations where the condition labels are randomly reassigned, and the whole procedure of cluster identification is repeated. The clusters in the original data are then compared with the top tail of the reference distribution according to the predefined statistical threshold (typically, 5%). The permutation testing requires no assumption of normality and affords a correction for the multiple comparison problem by controlling the familywise error rate. For full details of the method, see Maris (2012).

The cluster-forming threshold was *P* < 0.05, and the permutation test threshold was set at *P* < 0.025 (because it is a 2-sided test). The number of permutations was set to 5,000, which tenders a lowest possible *P* value equal to 0.0004. Cluster statistics were computed using the “ft_freqstatistics” routine in the FieldTrip toolbox. For testing of the effect of conditioning on the NPD estimate, statistics are computed identically as described above, but the conditioned and unconditioned spectra are treated as the two experimental conditions of interest. Because multiple recordings per subcortical site were obtained in each animal, we averaged the spectra from these recordings into a subject mean. Group-level plots indicate the group mean (bold curves) ± SE (shading).

## RESULTS

### Spectral Power

**Fig. 3.**
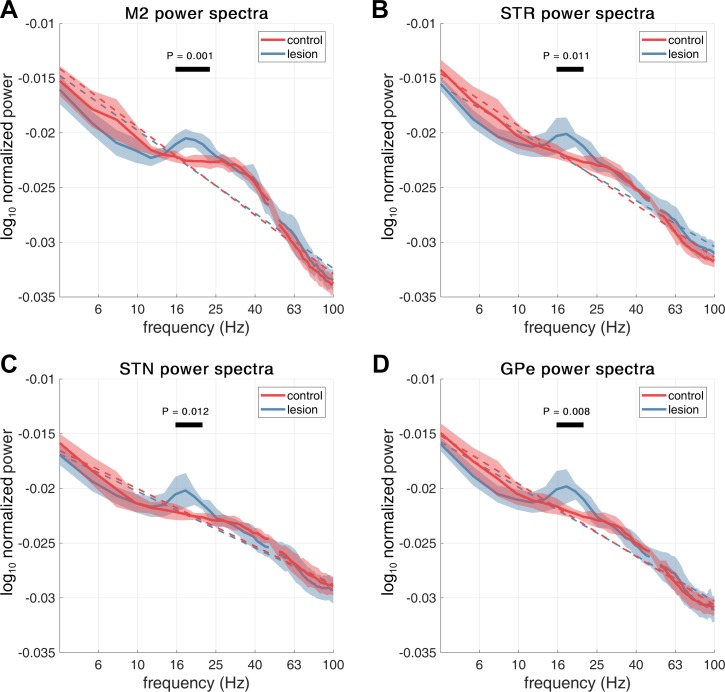
Group-averaged power spectra for all rats across both control and lesion conditions. Spectra are shown for signals recorded from M2 (*A*), STR (*B*), STN (*C*), and GPe (*D*). The group averages for the control and 6-OHDA dopamine depleted animals are shown by the red or blue lines, respectively. Shading shows the mean ± SE. Results of cluster permutation tests for the effect of the lesion are indicated by a black bar and corresponding *P* value. All recording sites presented beta peaks around 18–20 Hz. Cluster-based permutation testing for significant differences between conditions showed that there was a significant increase in beta in the lesioned animals for signals at all recorded sites. The dashed lines indicate a linear regression in log-log space as a rough estimate to the 1/*f* background.

Examples of spectra computed from LFP and ECoG signals recorded in individual animals are shown in Fig. 2, *B* and *D*. All the 6-OHDA-lesioned rats demonstrated a clear peak in the spectra in the range 18–22 Hz (encompassing low-beta/lower end of high-beta frequencies) for LFP recordings across all subcortical recording sites as well as for the sensorimotor ECoGs. In some animals, cortical beta was weaker than that observed subcortically. None of the LFP data from control animals contained beta peaks in the spectra, although some (4 of 8) showed a wide, low-amplitude peak around 20–40 Hz that was clearly above the 1/*f* background and most prominent in the recordings at M2 (an example of which is shown in Fig. 2*B*). Analysis of the group-averaged spectra (Fig. 3) shows that the beta peak is significantly increased in the dopamine-depleted animals. Cluster-based permutation testing demonstrated significant differences in group-level spectra between control and lesion conditions with clusters showing increases in power associated with dopamine depletion in the M2 (16–23 Hz, *P* = 0.001), STR (18–21 Hz, *P* = 0.011), STN (16–21 Hz, *P* = 0.012), and GPe (17–22 Hz, *P* = 0.008). No differences between lesioned and control animals were found for frequencies >22 Hz in any structures.

### Functional Connectivity: Imaginary Coherence

**Fig. 4.**
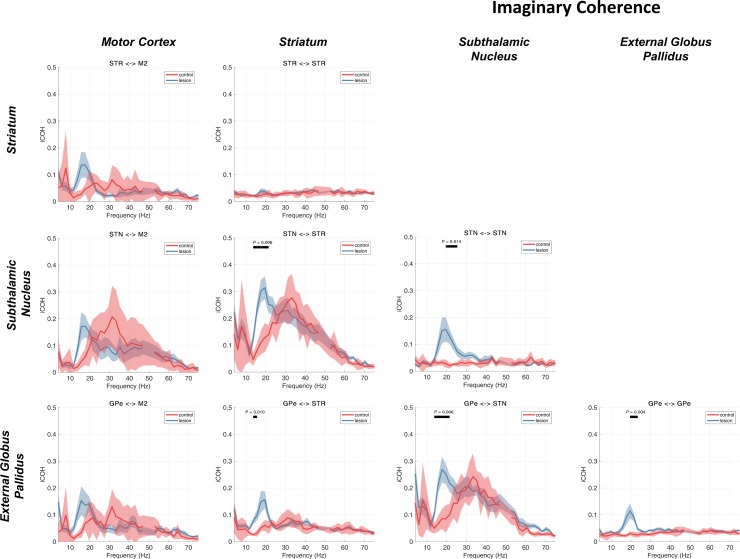
Functional connectivity estimates using imaginary part of coherence (iCOH). Group averages for the control and 6-OHDA dopamine depleted animals are shown by the red or blue lines, respectively. Shading shows the mean ± SE. Cluster-based permutation statistics were applied to test the effect of the lesion. Significant clusters are indicated by black bars above the spectra and corresponding *P* values. The iCOH metric, robust to zero-lag interactions, presents a richer view of functional connectivity that would otherwise be missed if standard coherence were used (data not shown). Beta activity is predominant across all cross-regional pairings. STN and GPe also show intranuclear correlations in this range in the dopamine-depleted state. Notably, there is also a high-beta/gamma interaction between STN/M2 and STN/STR that is visible in both control and lesioned animals.

Initial analyses of connectivity of the recorded LFPs using magnitude-squared coherence showed large-magnitude (>0.9) wideband (0–60 Hz) coherences that were indicative of a large field-spread effect (data not shown). This was most apparent in subcortical-subcortical analyses but was also detected for cortical-subcortical pairings. To estimate coherence while avoiding contamination by volume conduction, we opted to calculate non-zero phase-lag correlations using the imaginary part of coherence (iCOH; see Fig. 4).

We found that activity in the low beta range (14–20 Hz) associated with 6-OHDA dopamine depletion is spread diffusely across the network with all interregional comparisons showing a significant beta peak in the iCOH spectrum. Notably, the strongest coherence in the low beta band involved STN, with STN/STR and STN/GPe pairs both showing coefficients >0.2. Within-region connectivity (i.e., STN *contact 1* to *contact 2*) was found to be present in this frequency range only for recordings within STN or GPe, where there was a clear beta peak. No within-region connectivity was found in the STR, where the iCOH spectra were flat.

Analysis of statistical differences using the cluster-based permutation testing between control and lesioned animals showed significant increases of iCOH in the beta band in the lesioned animals and for 5/10 LFP pairs tested: STN/STR (14–21 Hz, *P* = 0.006), STN/STN (19–25 Hz, *P* = 0.014), GPe/STR (14–16 Hz, *P* = 0.010), GPe/STN (14–21 Hz, *P* = 0.006), and GPe/GPe (19–23 Hz, *P* = 0.004). Notably, no pairs involving M2 showed significant modulation of beta-band activity following dopamine depletion when tested using cluster statistics. Taken generally, these results are indicative of widespread non-zero-lag, low-beta-band connectivity across the entire cortico-BG network that is increased in the dopamine-depleted rats.

In the control rats, connectivity in the beta range was reduced relative to the dopamine depleted rats. Instead, there was wideband iCOH in the high-beta/low-gamma bands, ranging from 20 to 50 Hz in most cases but up to 70 Hz for the STN/M2 interactions. The majority of gamma-band interactions where iCOH was high (>0.2) were found in connections involving the STN. Additionally, iCOH in these bands is evident between GPe/M2 and GPe/STR, although this was weaker (at ~0.1) than connections analyzed with pairs involving the STN. iCOH in these bands is present in both the lesioned and control animals and does not show a strong modulation by dopamine, as evidenced by the lack of significant clusters in the permutation tests for these bands. The iCOH analyses present evidence for strong non-zero coherences at these frequencies even when spectral power at these frequencies is small. It should be noted that the oscillatory power of a signal is a separable, but complementary, property from that of the degree of correlation between two oscillatory signals such as that measured with coherence.

### Nonparametric Directionality

**Fig. 5.**
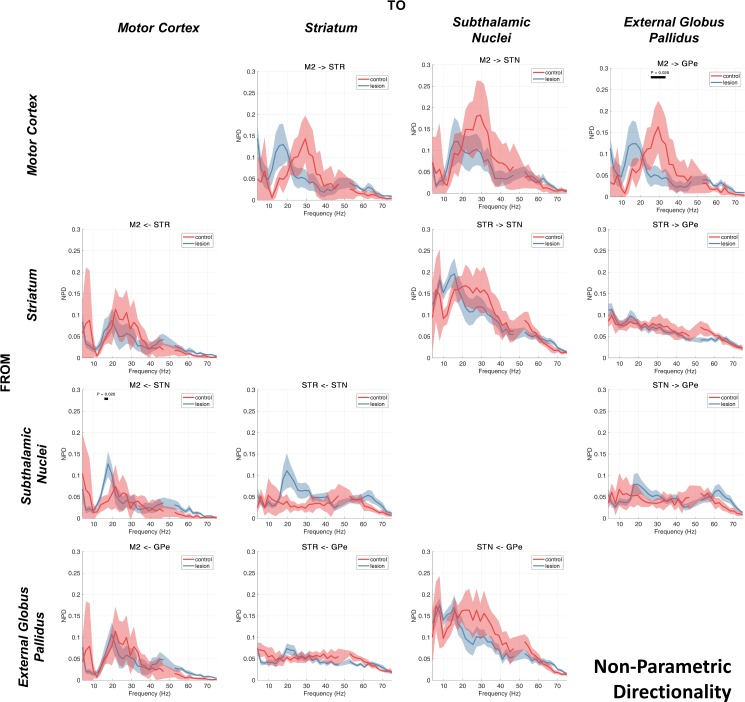
Directed connectivity estimated using nonparametric directionality (NPD) between subcortically recorded LFPs (GPe, STN, and STR) and ECoG recorded at M2. NPD decomposes the coherence between pairs of signals into forward and reverse components. The array of spectra is read such that each row shows the forward coherence of the indicated structure targeted to the structure indicated above each column. The group averages for the control and 6-OHDA dopamine depleted animals are shown by the red or blue lines, respectively. Shading shows the mean ± SE. Cluster-based permutation statistics were applied to test the effect of the lesion. Significant clusters are indicated by black bars above the spectra and corresponding *P* values.

We next investigated directed connectivity between recorded regions. The results of the analysis using the NPD measure are presented in Fig. 5. The iCOH and the sum of the noninstantaneous parts (forward and backward) of the NPD are similar, and both methods revealed similar patterns of connectivity (data not shown). Analysis of the instantaneous (zero lag) NPD in isolation demonstrated the existence of high-amplitude, wideband interactions that were similar to those found with magnitude-squared coherence (data not shown) and are likely due to zero-phase field spread of activity between recordings. Analyses of directional interactions of the LFPs and ECoG described from this point on use the forward and backward components of the NPD to discern directional connectivity between LFPs recorded from each brain structure. Investigation of individual animals’ functional connectivity revealed that for the majority of animals, the NPD spectra (and subsequently partialized spectra) corresponded well to that indicated by the group average.

We observed that directional interactions of low-beta-band activity in the dopamine-depleted animals predominate in the direction leading from M2 and that they descend the hierarchy of the BG. Interestingly, we noted a significant difference in the cortical-subthalamic beta-band interaction between lesioned and control animals only in the feedback connection STN → M2 (16–18 Hz, *P* = 0.020), which would suggest that STN feedback to M2 is strengthened in the dopamine-depleted state. In the case of the STN/GPe circuit, and unlike iCOH, the noninstantaneous components of NPD do not show 6-OHDA-related increases in beta coupling in either direction for the lesioned rats. Rather, NPD suggests a directional asymmetry in activity in the high-beta/gamma band with forward connections GPe → STN stronger than in the reverse direction (cluster statistics testing differences between forward and backward spectra in the 6-OHDA recordings: 4–43 Hz, *P* < 0.001). Notably, we see a feedback in STN → STR that is most prominent in the lesion condition, a feature that will be relevant when taken into account with further results presented in the section *Inferring Routing of Brain Rhythms: Partialized NPD*.

The pattern of activity in the high-beta/gamma range between cortical and subcortical regions appeared to be principally cortically leading with the coefficient of the interactions in the 20- to 40-Hz range being up to two-thirds larger in the dopamine-intact control rats (Fig. 5, 1st row). Cluster-based permutation analysis showed a significant increase in the high-beta/gamma M2 → GPe NPD in the control vs. the lesion condition (25–30 Hz, *P* = 0.020). High-beta/gamma connections from subcortical structures feeding back to M2 are weaker than the cortically leading connections but are still present for striatal and globus pallidus feedback to M2 (Fig. 5, 1st column, 2nd and 4th rows). Again, there was a clear peak in the high-beta NPD from STN→ STR in the lesioned animals, although a dependence on dopamine was not seen to be significant during testing with cluster statistics. The finding of a large NPD interaction from STN to STR does not accord with the canonical circuit (Fig. 1*A*) but may instead imply feedback to striatum via subcortical thalamo-striatal loops. This is discussed in hypothesis 6: high-beta/gamma is generated locally within the basal ganglia network at str, stn, or gpe.

### Inferring Routing of Brain Rhythms: Partialized NPD

We repeated the NPD analysis as before but this time systematically partializing out (conditioning) the contribution made by LFPs/ECoG recorded from each brain structure to the bivariate analyses presented in results, *Nonparametric Directionality*. We again employed cluster statistics to determine significant differences between the nonconditioned NPD spectra and its conditioned variant that is presented in the results shown below.

#### Conditioning the NPD using LFPs recorded from the STN.

**Fig. 6.**
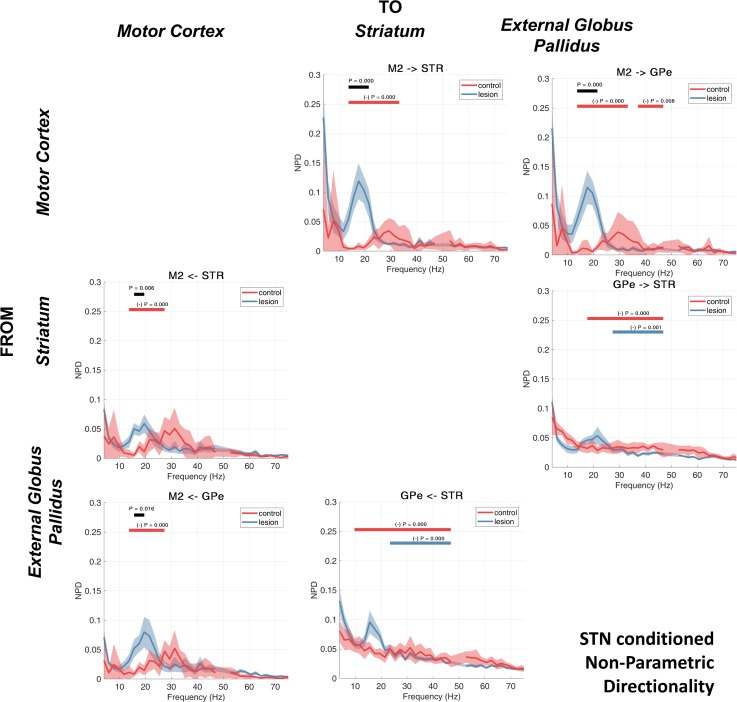
NPD conditioned on the STN LFP. Group averages for the control and 6-OHDA dopamine depleted animals are shown by the red or blue lines, respectively. Shading shows the mean ± SE. Cluster-based permutation statistics were applied to test the effect of the lesion. Significant clusters are indicated by black bars above the spectra and corresponding *P* values. The effect of conditioning with the STN LFP was also tested using cluster permutation statistics. Frequencies where NPD was significantly attenuated by the conditioning are indicated by red and blue bars (and corresponding *P* values) for the control and lesion recordings, respectively.

We first conducted a partialization (conditioning) of the NPD estimate using LFPs recorded from within the STN (Fig. 6). Conditioning with signals from the STN does not remove beta connectivity between the remaining structures in the network, although it does weaken the majority of comparisons in the control (6 of 6 comparisons; Fig. 6, red bars) but not the lesion animals (2 of 6 comparisons, Fig. 6, blue bars). Cluster statistics indicate that the following NPDs for the control experiments were significantly reduced by conditioning with the STN signal: M2 → STR (14–33 Hz, *P* < 0.001), M2 → GPe (14–33 Hz, *P* < 0.001; 37–49 Hz, *P* = 0.008), STR → GPe (10–49 Hz, *P* < 0.001), and GPe → STR (18–49 Hz, *P* < 0.001), as well as feedback connections (returning to cortex): STR → M2 (14–27 Hz, *P* < 0.001) and GPe → M2 (18–49 Hz, *P* < 0.001). Furthermore, conditioning the NPD with the signal from STN does not disrupt the 6-OHDA-associated increases of M2 input to either the STR (14–21 Hz, *P* < 0.001) or GPe (14–21 Hz, *P* < 0.001) (Fig. 6, black bars). We also found in the dopamine-depleted state that there was increased (relative to controls) feedback to M2 from both GPe (16–20 Hz, *P* = 0.016) and STR (16–20 Hz, *P* = 0.006).

Notably, we observed some separation in the effects of the conditioning between the control and lesion experiments. In the control animals, conditioning the NPD on LFPs recorded at STN acted to reduce activity in a wide band (~12–40 Hz) for the forward connections (propagating down the indirect pathway; i.e., M2 → STR, M2 → GPe, and STR → GPe), whereas the return connections (STR → M2 and GPe → M2) were only affected by conditioning at a tighter band corresponding to low beta. This would suggest that in the healthy animal, signals returning to cortex via STN occur at low-beta frequencies. Lesioned animals only showed reductions at higher frequencies (~24–45 Hz, high beta/low gamma), and only between GPe and STR. We observed that conditioning of the NPD with the STN signal acted to significantly reduce interactions between STR and GPe in both the forward (STR → GPe, 23–49 Hz, *P* < 0.001) and reverse (GPe → STR, 27–49 Hz, *P* = 0.001) directions (Fig. 6, red bars).

#### Conditioning the NPD using LFPs recorded from the GPe.

**Fig. 7.**
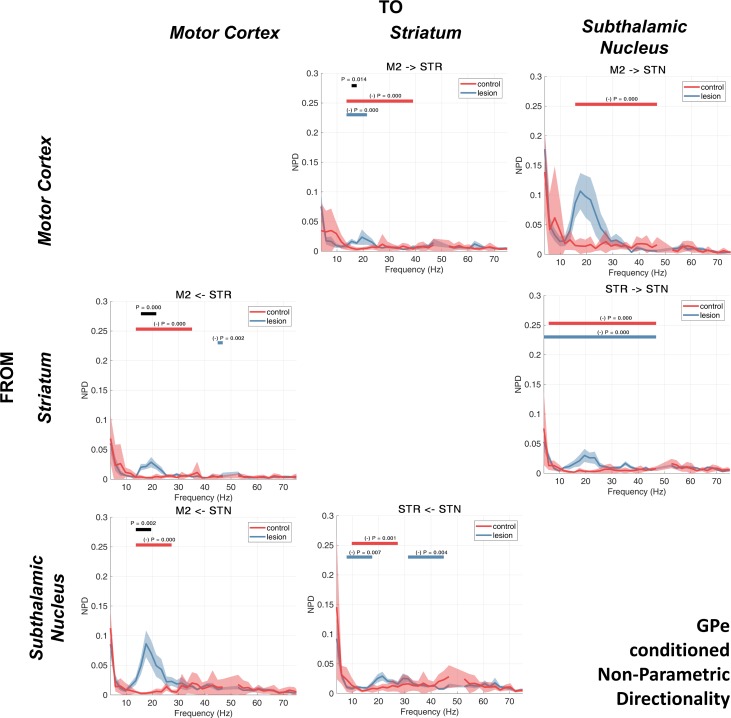
NPD conditioned on the GPe LFP. Group averages for the control and 6-OHDA dopamine depleted animals are shown by the red or blue lines, respectively. Shading shows the mean ± SE. Cluster-based permutation statistics were applied to test the effect of the lesion. Significant clusters are indicated by black bars above the spectra and corresponding *P* values. The effect of conditioning with the GPe LFP was also tested using cluster permutation statistics. Frequencies where NPD was significantly attenuated by the conditioning are indicated by red and blue bars (and corresponding *P* values) for the control and lesion recordings, respectively.

Next, we performed the NPD analysis of recorded signals, this time conditioning the interactions with LFPs recorded from within the GPe (Fig. 7). We found that the conditioning had the effect of reducing NPD estimates in 6 of 6 possible connections in the controls and 3 of 6 in the 6-OHDA-lesioned rats. Most notably, we found that the conditioning significantly attenuated (compared with the unconditioned NPD) the low-beta-band interaction in the M2 → STR connection for recordings made in both control (14–39 Hz, *P* < 0.001; Fig. 7, red bar) and lesioned animals (14–21 Hz, *P* < 0.001; Fig. 7, blue bar), implying that signals propagating through STR are highly correlated with those also measured at GPe.

We found a reduction of interactions between STR → STN across a wide range of frequencies, again for recordings in both control (6–49 Hz, *P* < 0.001; Fig. 7, red bar) and lesioned animals (4–49 Hz, *P* < 0.001; Fig. 7, blue bar), suggesting signal routing is strongly mediated by GPe in accordance with the canonical indirect pathway. Interestingly, we found that although beta NPD in the M2 → STN connection was attenuated by conditioning in the control recordings: for the 6-OHDA recordings, the prominent low-beta peak in the NPD remained, and no significant effect of conditioning was observed. Similarly, the STN → M2 feedback also retained a sharp beta peak that remained significantly increased in recordings corresponding to the 6-OHDA lesion experiments (14–20 Hz, *P* = 0.002; Fig. 7, black bar). Additionally, we found that when the STR→ M2 NPD estimate was conditioned with the GPe signal, there was an increased strength of interaction in the 6-OHDA-treated animals (16–21 Hz, *P* < 0.001; Fig. 7, black bar).

In the high-beta/gamma band, we found that conditioning with GPe had a large effect in attenuating the NPD in the forward connections (from M2 descending the indirect pathway) in the control animals: M2 → STR (14–39 Hz, *P* < 0.001), M2 → STN (16–49 Hz, *P* < 0.001), and STR → STN (6–49 Hz, *P* < 0.001) (Fig. 7, red bars). In the lesioned animals only, 2 of the 6 comparisons made with NPD were significantly attenuated in the 20- to 50-Hz range: STR → STN (4–49 Hz, *P* < 0.001) and STN → STR (31–45 Hz, *P* = 0.004) (Fig. 7, blue bars). This would imply that in control animals, high-beta/gamma-band interactions in both directions between STN and STR are transmitted via (and linearly mixed with) a signal at GPe.

#### Conditioning the NPD using LFPs recorded from the STR.

**Fig. 8.**
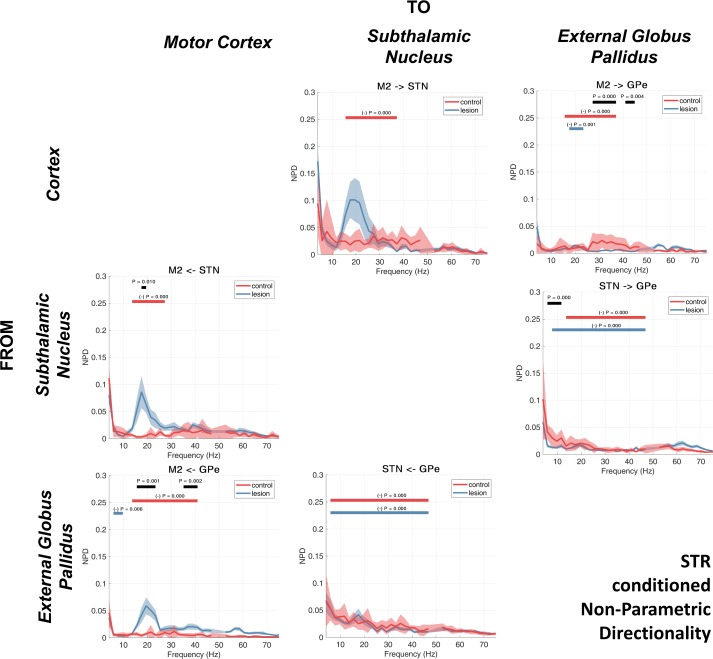
NPD conditioned on the STR LFP. Group averages for the control and 6-OHDA dopamine depleted animals are shown by the red or blue lines, respectively. Shading shows the mean ± 1 SE. Cluster-based permutation statistics were applied to test the effect of the lesion. Significant clusters are indicated by black bars above the spectra and corresponding *P* values. The effect of conditioning with the STR LFP was also tested using cluster permutation statistics. Frequencies where NPD was significantly attenuated by the conditioning are indicated by red and blue bars (and corresponding *P* values) for the control and lesion recordings, respectively.

A third set of analyses used the LFPs recorded at the STR to condition the NPD estimates (Fig. 8). We found that this had the effect of destroying large parts of the descending interactions (connections from M2 descending the hierarchy of the indirect pathway) in the control animals, namely, for M2 → GPe (16–37 Hz, *P* < 0.001) and M2 → STN (16–37 Hz, *P* < 0.001) (Fig. 8, red bars). In the lesion recordings, the effect of conditioning split two ways: *1*) interactions between the STN/GPe were significantly reduced across a very wide band ranging from low-beta to gamma frequencies in both the STN → GPe (8–49 Hz, *P* < 0.001) and GPe → STN (6–49 Hz, *P* < 0.001) couplings (Fig. 8, blue bars), and *2*) interactions in the hyperdirect M2 → STN connection were not attenuated, although note that M2 → GPe (likely routed at least in part via the indirect pathway) was suppressed by conditioning with the striatal signal (18–24 Hz, *P* = 0.001; Fig. 8, blue bar). This peak is also seen in the feedback connection from STN → M2 where the significant 6-OHDA-associated increase in beta feedback reported in previous analysis was found to remain (18–20 Hz, *P* = 0.010; Fig. 8, black bar).

Similar to the NPD estimates conditioned with signals recorded at GPe, we found that conditioning with LFPs recorded at STR acted to largely remove the high-beta/gamma interactions. In the M2 → GPe connection in control animals, we found that high-beta/gamma activity was attenuated by STR conditioning (16–37 Hz, *P* < 0.001); furthermore, we observed that 6-OHDA was associated with a significant suppression of activity in this band (27–37 Hz, *P* < 0.001 and 41–45 Hz, *P* = 0.004). Additionally, we found that feedback in the high-beta/gamma range (for control recordings) from GPe → M2 was significantly attenuated by conditioning with the signal recorded at STR (14–41 Hz, *P* < 0.001; Fig. 8, red bar). Furthermore, this connection from GPe → M2, was significantly strengthened in the 6-OHDA animals (35–41 Hz, *P* = 0.002; Fig. 8, black bar).

#### Conditioning NPD using ECoG recorded from M2.

**Fig. 9.**
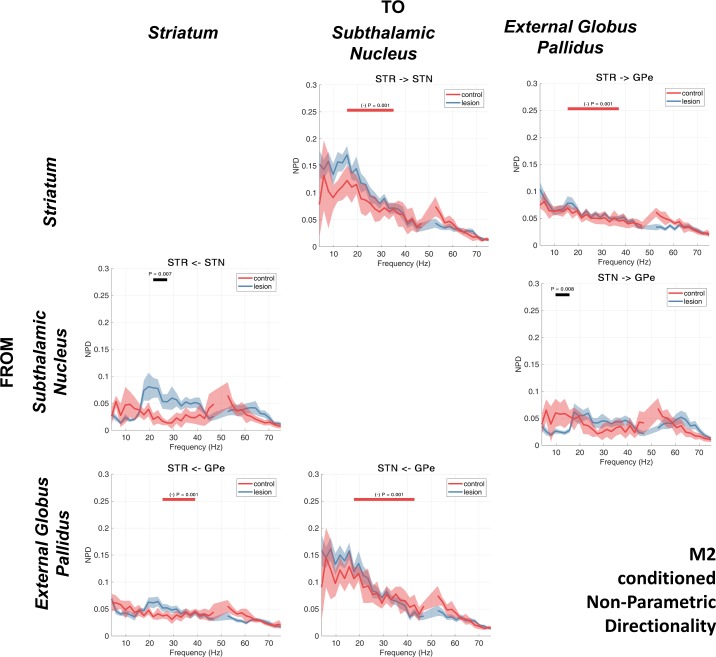
NPD conditioned on the M2 electrocorticogram. Group averages for the control and 6-OHDA dopamine depleted animals are shown by the red or blue lines, respectively. Shading shows the mean ± SE. Cluster-based permutation statistics were applied to test the effect of the lesion. Significant clusters are indicated by black bars above the spectra and corresponding *P* value. The effect of conditioning with the M2 ECoG was also tested using cluster permutation statistics. Frequencies where NPD was significantly attenuated by the conditioning are indicated by red and blue bars (and corresponding *P* values) for the control and lesion recordings, respectively.

The final analyses utilized ECoG signals recorded from the M2 to condition the BG NPD estimates (Fig. 9). We found that the NPD estimates conditioned on M2 were generally flattened and lacked distinct peaks at either low-beta or high-beta/gamma frequencies that were seen typically in the other analyses. Altogether, 5 of 6 NPD spectra had no distinct spectral peaks. When testing for significant attenuation of NPD following conditioning, we found that only control recordings were significantly attenuated (4 of 6 connections; Fig. 9, red bars), with high-beta/gamma peaks most clearly lost in the STR→ STN and STN → GPe interactions. The loss of features found in the unconditioned NPD (such as beta or gamma peaks) was equivalent for both the control and 6-OHDA recordings.

When testing for the effects of 6-OHDA, we found that the STN → STR connection was significantly altered. We observed a broad peak from 20 to 40 Hz in the lesion recordings that was not attenuated by M2 conditioning and demonstrated a significant increase in strength associated with dopamine depletion (21–27 Hz, *P* = 0.007; Fig. 9, black bar).

### Summary of Connectivity Analyses

Using recordings made in control and lesioned rats, we identified functional connectivity between cortical and BG sites that involved either low-beta or high-beta/gamma oscillations. Broadly speaking, we found that gamma connectivity is sensitive to the conditioning of structures upstream of the STN, particularly GPe and STR, which removes gamma-band oscillations from the spectra. In contrast, beta connectivity was found to be robust to partializing by using LFPs of any single BG structure. Cortico-subthalamic connectivity in the beta range was unaffected by partializing of GPe or STR, suggesting that M2/STN low-beta connectivity is not routed via the indirect pathway. In the discussion, we outline several putative models of oscillatory dynamics and present evidence from our analyses that either support or weaken the plausibility of each model.

## DISCUSSION

### Hypotheses and Evaluation of Evidence for Signal Propagation in the Network

We have undertaken a systematic analysis of data from an experiment involving multisite ECoG/LFP recordings of the cortico-basal ganglia circuit that contains signals from a group of dopamine-intact control rats and a separate group of rats with chronic dopamine depletion induced by a unilateral injection of 6-OHDA. Below we discuss evidence for competing theories of the propagation of oscillatory activity across the parkinsonian cortico-basal ganglia circuit. We emphasize that our results are indicative of the transmission of rhythmic activity in the circuit and cannot directly access the mechanisms that generate these rhythms. However, as we argue below, results describing the patterns of synchronized activity across the network and the changes that occur to them following dopamine depletion proffer an important insight into how pathological rhythms differentially engage functional networks.

#### Mechanisms of the flow of beta rhythms in the basal ganglia circuit.

**Fig. 10.**
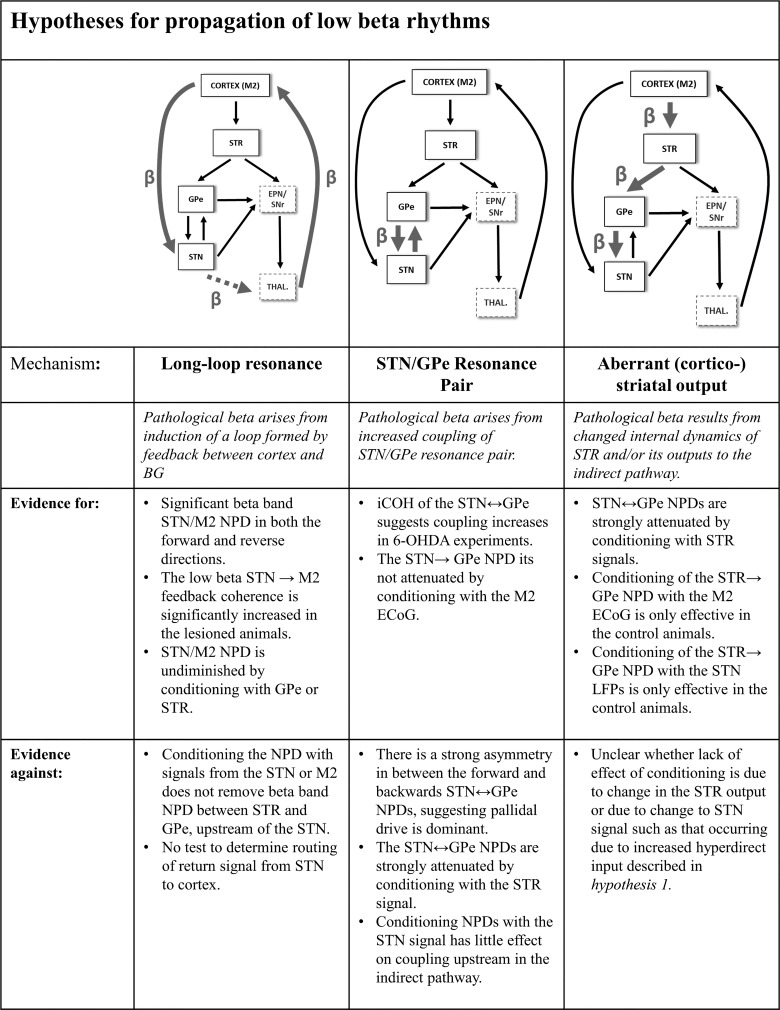
Summary of hypotheses of the impact of dopamine depletion on the propagation of beta rhythms in the cortico-basal ganglia circuit.

Below we evaluate the evidence provided by the analyses reported in this study in light of a number of proposed theories concerning the generation and propagation of beta-band activity in the network and the changes that occur during dopamine depletion that lead to its amplification. This body of work is summarized in Fig. 10.

##### hypothesis 1: dopamine depletion in the basal ganglia induces increased beta resonance in the cortical/stn “long loop.”

Previous authors have suggested that pathological beta rhythms are generated from the strengthening of a long cortical feedback loop that returns from BG output nuclei via the thalamus. Strengthened coupling is proposed to facilitate pathological resonance at beta frequencies (Brown 2007; Dovzhenok and Rubchinsky 2012; Pavlides et al. 2015; van Albada and Robinson 2009). The first step toward verifying the plausibility of this hypothesis involves determining whether there is indeed functional connectivity between STN and M2 in the beta band, and whether this occurs independently of the corticostriatal inputs to the indirect pathway.

Analysis of the iCOH for the M2/STN pairing suggests that functional connectivity in the beta band is significantly strengthened in the lesioned animals compared with controls (Fig. 4). Analysis with NPD demonstrates that there is a beta peak in the directed coherence in the low-beta range in the forward M2 → STN connection for both the control and 6-OHDA-lesioned animals. Furthermore, in the lesioned animals, the feedback connection (STN → M2) is significantly strengthened over that measured in the controls. Neither the hyperdirect M2 → STN connection nor the subthalamo-cortical feedback (STN → M2) is diminished by conditioning with signals from either the GPe or STR in the lesioned animals (Figs. 7 and 8). This suggests a reciprocal pathway between STN and M2 that is routed independently of STR or GPe, most likely feeding back directly via the BG output nuclei. In contrast, in control rats, NPD of the feedback connections at beta frequencies are significantly decreased by conditioning with the STR signal in the forward (M2 → STN) and backward (STN → M2) directions, suggesting that in the dopamine-intact anesthetized state, beta-band activity is routed via STR, whereas the hyperdirect pathway is relatively quiescent. These findings support the idea that the dopamine-depleted state is associated with a strengthening of the hyperdirect pathway and subthalamo-cortical feedbacks.

Notably, this pathway is not active in isolation but coexists with beta propagation occurring along striatal indirect pathway projections. Most notably, it was found that conditioning of the NPD with LFPs recorded from the STN (Fig. 6) does not act to remove the 6-OHDA lesion-associated beta NPD in the structures “upstream” of the STN (i.e., the STR and GPe). NPD in the low-beta range is significant in both directions along parts of the network involving either M2, STR, or GPe. We find that striatal-subthalamic interactions are strongly modulated by the GPe signal, a finding in line with propagation down the canonical indirect pathway. Future work to validate the long-loop hypothesis would involve the conditioning of the STN → M2 NPD using signals recorded from BG output nuclei [either internal globus pallidus (GPi/EPN in rat) and/or substantia nigra pars reticulata (SNr)] or their major targets in the thalamus. If these signals were available, then it would be possible to better determine the routing of the cortical return of BG beta activity from the STN.

##### hypothesis 2: pathological beta is generated from strengthening of the reciprocally coupled stn/gpe circuit.

A separate hypothesis concerning the generation of pathological beta rhythms in the BG considers the reciprocally coupled STN/GPe circuit from which an increase in coupling associated with the loss of dopamine induces a pathological beta resonance that spreads across the rest of the network (Bevan et al. 2002; Holgado et al. 2010; Plenz and Kital 1999; Tachibana et al. 2011).

We note that conditioning the NPD with the M2 signal does not remove the strong STN → GPe directed connectivity, but it does attenuate the GPe → STN connection (Fig. 9). This indicates that activity feeding back onto GPe from STN has a sufficiently unique temporal content so as not to be partialized out by the cortical ECoG, suggesting that pathological beta activity could be generated by some resonance phenomenon arising from the tight, reciprocal coupling of STN and GPe. However, a number of the analyses presented in this article suggest that pathological beta does not originate from an autonomous STN/GPe resonator. These can be summarized as follows: *1*) comparison of forward and backward NPD for STN/GPe interactions shows strong asymmetry, with the GPe→ STN connection predominating; *2*) conditioning of the NPD using the LFPs recorded at the STR significantly reduces the strength of both GPe → STN and STN → GPe NPDs in a way that appears to be irrespective of dopaminergic state (Fig. 8), suggesting that beta activity in these structures results from beta oscillations propagating through striatum; *3*) conditioning the NPD with LFPs recorded at the STN (Fig. 6) does not act to remove the upstream 6-OHDA-associated beta NPD between STR or GPe (although it does significantly weaken beta NPD in the control animals); *4*) GPe-conditioned NPD analysis does not diminish the pathological M2/STN beta interactions (Fig. 7), suggesting that the beta found at STN can be, at least in part, generated independently of a signal found at GPe. The evidence given in *item 1* may arise from the very tight coupling of the STN/GPe pair: if full phase synchronization is occurring, then the phase alignment between the two nuclei may mislead the NPD to determine the phase-leading population to be the drive, when in actuality there is strong reciprocal coupling. The evidence in *items 2* and *3* point toward a mechanism of striatal modulation of the STN/GPe circuit, perhaps via a pallidal-striatal feedback mechanism such as that described by Corbit et al. (2016). When taken together, we argue these findings provide evidence against pathological beta synchronization in the network arising from dissemination of an autonomously generated rhythm in a STN/GPe “resonator.”

##### hypothesis 3: beta arises through aberrant striatal activity and facilitation of downstream hypersynchrony.

It has been proposed that aberrant striatal activity is involved in the emergence of pathological beta rhythms in the BG arises due to changes to local dynamics within striatum (Damodaran et al. 2015; McCarthy et al. 2011; Sharott et al. 2017) and/or a modification of striatal influence on the STN/GPe subcircuit (Kumar et al. 2011; Sharott et al. 2017; Terman et al. 2002). From iCOH analysis of signals recorded within striatum, we do not find any local non-zero phase interactions (unlike that which we find at STN). This finding would suggest that striatal-striatal transmission is sparse, or phase aligned. Our results show that NPD measured at both the STN and GPe are significantly weakened by conditioning with STR signals (Fig. 8), implying that striatal beta-band activity propagates down the indirect pathway. This would be in line with the recent demonstration that the firing of indirect pathway spiny projection neurons is aberrantly (and selectively) entrained to exaggerated beta oscillations in lesioned rats (Sharott et al. 2017).

The weakening of NPD interactions from STR → GPe and GPe→ STN during conditioning with M2 ECoG (Fig. 9), and only for the dopamine intact controls, may suggest that dopamine depletion results in increased autonomy of the striatal (and indirect pathway) beta rhythm from beta at M2. In support of this hypothesis, we also demonstrate that conditioning of the STR → GPe NPD with the STN signal is only effective (within the low-beta range) in the control condition. This again demonstrates that 6-OHDA lesioning results in a striatal signal that retains information independent from that found at STN, providing evidence that it is likely the change of striatal output that occurs following dopamine depletion. There is, however, some ambiguity as to whether the separation of the striatal signal from that at the STN occurs due to changes in striatal dynamics or, instead, due to a change of direct input to the STN such as from a strengthened hyperdirect input as discussed in *hypothesis 1*.

#### Hypotheses of the origins/routing of high-beta/gamma oscillations.

**Fig. 11.**
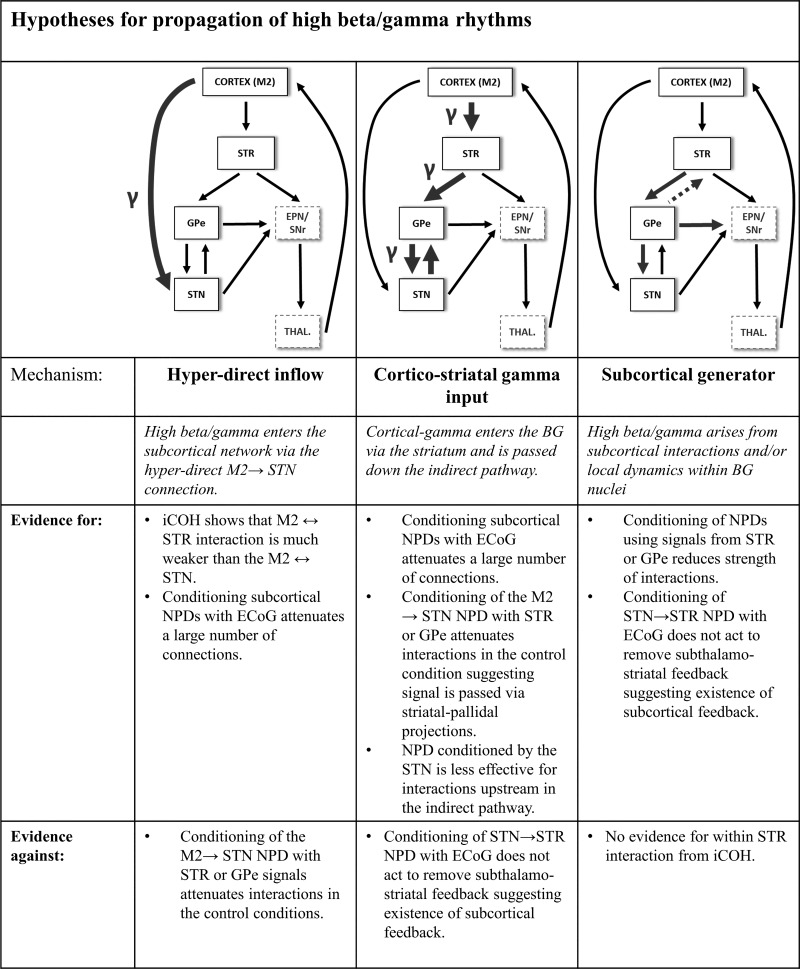
Summary of hypotheses for gamma flow in the cortico-basal ganglia circuit.

The presence of high-beta/gamma oscillations in the subcortical network has been noted by a number of authors (Berke 2009; Brown et al. 2002; Humphries et al. 2006; Nicolás et al. 2011; Sharott et al. 2009; van der Meer et al. 2010), but our understanding of the functional propagation of high-beta/gamma oscillations through the network is limited. An evaluation of the evidence we present in this article is summarized in Fig. 11. We report gamma activity in the LFPs as well as connectivity in the range 30–60 Hz, which is in good agreement with that previously reported in anesthetized rats (Magill et al. 2004; Sharott et al. 2005b, 2009). Gamma activity in the awake and moving rat has also been reported, albeit at slightly higher frequencies (Brazhnik et al. 2012; Brown et al. 2002; Delaville et al. 2014).

##### hypothesis 4: high-beta/gamma rhythms enter the subcortical network via the hyperdirect m2 → stn connection.

Results from analyses for which we used iCOH to investigate non-zero lag correlations between BG structures and the cortex suggest that gamma interactions are routed in a way that bypasses STR, because a gamma peak is absent in the M2/STR iCOH (Fig. 4). This effect is most clear in the control recordings but also to a lesser extent in the 6-OHDA experiments. The hyperdirect pathway is the other principal source of cortical input to the BG; therefore, the marked weakness of gamma interaction in the M2/STR compared with the M2/STN iCOH spectra may imply that the hyperdirect pathway is responsible for gamma input to the network.

However, although there is a large peak in the high-beta/low-gamma-band NPD for the M2 → STN interaction (Fig. 5), if we examine the same connection but conditioned on LFPs recorded at either STR (Fig. 7) or GPe (Fig. 8), we see that the conditioning significantly reduces NPD in the control animals (M2 → STN conditioned on STR and M2 → STN conditioned on GPe), suggesting any directed coherence between M2 and STN in these animals is routed via striatal-pallidal connections. Furthermore, if we condition the NPD with LFPs recorded at the STN (Fig. 6), we see that gamma interactions remain in the upstream components (M2 → STR, M2 → GPe), again suggesting striatal-pallidal connectivity is vital in the propagation of gamma rhythms. When taken together, these data do not supply strong evidence that the source of high-beta/gamma input in the network is transferred by a hyperdirect cortico-subthalamic route.

##### hypothesis 5: gamma rhythms enter the network via cortico-striatal inputs and reach stn via the indirect pathway in a dopamine-dependent manner.

An alternative to high-beta/gamma oscillations entering via hyperdirect STN input is that they are channeled via the cortico-striatal indirect pathway. The clearest results of the NPD analysis in the high-beta/gamma band can be seen to be for the forward NPDs originating from M2 and passing on to the subcortical regions (Fig. 5). Connections M2 → STR, M2 → GPe, and M2 → STN all show high values of NPD in this frequency band (>0.15), suggesting that most of the gamma activity is directed from the cortex. Furthermore, conditioning the NPD with LFPs recorded at either the STR (Fig. 8) or GPe (Fig. 7) acts to remove gamma interactions both upstream and downstream of the STR (with respect to the indirect pathway). Subsequently, conditioning of the NPD with STN (Fig. 6) is less effective at attenuating gamma-band interactions than when signals higher in the indirect pathway are used, suggesting that the gamma rhythm descends the hierarchy, from either a cortical or striatal source. Notably, we observed that STN-conditioned NPD did not act to attenuate feedback connections from GPe or STR back to M2. This would suggest routing of gamma activity to M2 in a way that occurs independently of STN.

In attempt to elucidate the source of the gamma activity, we conditioned the NPD on the cortical ECoG (Fig. 9). We find that gamma connectivity in the control recordings and in dopamine depletion states acts to significantly reduce NPD coefficients for the GPe → STN and STR → STN connections, yet the feedback connection STN → STR is unaffected. This connection in the control animals shows a peak from 18 to 42 Hz that is significantly larger than in the lesioned animals. This is in agreement with the hypothesis that gamma rhythms are prokinetic; this idea is also supported by patient data (Sharott et al. 2014). Furthermore, these findings suggest that gamma activity is directed to upstream components of the indirect pathway in a way independent of M2, perhaps mediated via a subcortical feedback loop.

##### hypothesis 6: high-beta/gamma oscillations are generated locally within the basal ganglia network at str, stn, or gpe.

The finding that conditioning the NPD with cortical ECoG does not entirely abolish gamma connectivity within the BG suggests a possible subcortical generator of high-beta/gamma oscillations or, alternatively, a source in the cortex that has not been measured in our experiments. Work by Kondabolu et al. (2016) has demonstrated that the optogenetic activation of striatal cholinergic interneurons is sufficient to generate gamma rhythms locally, although not in a way clearly separable from low-frequency beta. However, when applying iCOH to signals recorded within STR, we find no evidence for local interactions in the high-beta/gamma band. Simulations of the BG spiking network by Humphries et al. (2006) suggest that upper gamma-band (40–80 Hz) activity can arise as a result of coupling between the STN and GPe. When we conditioned the NPD with LFPs recorded from either the GPe (Fig. 7) or STR (Fig. 8), we found that interactions in the high-beta/gamma frequency ranges were abolished in the majority of other subcortical interactions. This would imply that these GPe and STR structures are necessary for the propagation of high-beta/gamma interactions in both the control and 6-OHDA-lesioned animals. This, in combination with the evidence provided for *hypothesis 5*, suggests that high-beta/gamma rhythms can originate at either STR or GPe and then propagate to downstream structures. Backward gamma interactions from GPe to STR are apparent in the NPD conditioned on either M2 or STN, suggesting the STR signal is the result of local propagation of a gamma signal from GPe. From the canonical circuit perspective, it is not clear how gamma activity passes upstream from GPe. However, a substantial proportion of GPe neurons that innervate the striatum have been shown to exist, with one GPe cell type (arkypallidal neurons) projecting exclusively to striatum (Abdi et al. 2015; Hegeman et al. 2016; Mallet et al. 2012). This same pathway has been proposed by Corbit et al. (2016) to promote synchronization in the low-beta frequency range, but the same arguments are likely to apply to high-beta/low-gamma frequencies.

### Summary of Findings

We have investigated the propagation of oscillatory activity through connected regions of the cortico-basal ganglia network. We have applied a novel model-free method of partialized directed connectivity to achieve a systematic deconstruction of the transmission of rhythmic activity between regions of the network inferred from the LFPs and ECoGs recorded at multiple sites within that network. Using the 6-OHDA-lesioned rat model of parkinsonism, we have demonstrated marked differences in the patterns of functional connectivity that result as a consequence of dopamine depletion in the BG.

We find widespread beta synchronization of LFPs across the network that is strongly associated with chronic dopamine depletion. With regard to functional beta connectivity in the network, we find evidence for

1. An increased cortical entrainment of the basal ganglia following dopamine depletion;
2. Significant beta-band connectivity between structures interacting with the STN that is independent of activity upstream in the indirect pathway (at STR and GPe) (this is likely to originate from the hyperdirect cortico-subthalamic projections);
3. Increase in feedback of BG structures to M2 after dopamine depletion, proffering evidence in favor of a hypothesis of dopamine-dependent modulation of the long reentrant cortico-BG-thalamo-cortical loop;
4. Activity within the STN/GPe subcircuit that is at least in part dependent on output from striatum; and
5. A feedback from STN to STR that is independent of M2 and significantly strengthened after dopamine depletion, suggesting a strengthening of recurrent subcortical circuits.

Furthermore, we provide evidence for the existence of high-beta/gamma synchrony within the network, with evidence that dopamine depletion acts to weaken these rhythms. We summarize our findings with respect to high-beta/gamma-band interactions in the following:

1. Gamma propagates down the indirect pathway from STR to GPe to STN. This activity is likely to be generated at the level of the cerebral cortex.
2. There is evidence of gamma activity found at STN that is independent of M2 and evidence for a subcortical return of subthalamic outputs back to striatum.
3. There is evidence of gamma activity returning to the cortex that is independent of STN, perhaps indicating propagation through the direct pathway.

#### Propagation of low-beta via two coexisting but distinct streams.

In the case of low-beta oscillations, our data most strongly support a hypothesis that in the dopamine-depleted condition, beta propagation in the network is biased to favor low-beta synchrony via induction of a long cortico-subthalamic loop that inputs to the BG via the hyperdirect pathway. Furthermore, we see evidence that the return connection from STN to M2 is significantly stronger in the lesioned animals compared with dopamine-intact controls. This provides supporting evidence for the notion that pathological beta amplification arises from entrainment of the reentrant cortical/STN loop (Brittain and Brown 2014). We speculate that strengthening of the hyperdirect input acts to “short-circuit” the network such that transmission of information along the indirect pathway is compromised. Oswal et al. (2016) have provided evidence that deep brain stimulation in patients acts to selectively suppress activity-mediated synchrony between mesial premotor regions and the STN that is proposed to be mediated by the hyperdirect pathway. In the “hold your horses” model of the STN’s role in decision making (Frank 2006; Frank et al. 2007), the hyperdirect pathway is proposed to provide a cortical veto signal that may act to suppress premature action. In the case of PD, overactivity of this circuit via increased resonance may act to lock the network into a state that ultimately suppresses action and movement. These findings are in agreement with previous research that has found good evidence for bidirectional connectivity between STN and cortex (Jávor-Duray et al. 2015; Lalo et al. 2008).

This hypothesis requires further testing through analysis of the role of the BG output nuclei at GPi or SNr (or their targets in the thalamus) in the propagation of activity. This could be achieved using a functional “lesion” approach like that described in this article. Furthermore, biophysical modeling of the cortico-subthalamic loop may yield insight as to whether this is a plausible mechanism given the known conduction delays for the connections in the network. Long feedback loops involving cortex have been demonstrated to be capable of generating oscillatory activity (Leblois et al. 2006; Pavlides et al. 2015). However, work by Shouno et al. (2017) suggests that the required delay for the return of the beta oscillation from STN to cortex may be too great to support resonance in the low-beta band, suggesting that the engagement of shorter subcortical relays, such as either the subcortical-thalamic loops (McHaffie et al. 2005) or activity of recurrent subthalamo-striatal projections (Koshimizu et al. 2013; Sato et al. 2000), may be more suitable candidates for supporting beta oscillations through resonance.

The analysis presented in this study also suggests that a cortico-subthalamic pathway is not the exclusive pathway for beta rhythms within the network yet may be necessary for enhancement of the STN feedback to cortex that may induce pathological resonance. We suggest that both the hyperdirect and indirect routes for beta propagation coexist. These two pathways could originate from, and be driven by, distinct populations of cortical projections neurons (namely, those of the pyramidal tract and intratelencephalic projections, respectively) and so are likely to show a degree of independence from one another. The present data also suggest a second pathway upstream of STN involving the STR that is most evident in the recordings from control rats. We suggest that both pathways contain signals shared by activity measured in the cortical ECoG: conditioning of the NPD acts to remove beta peaks from the majority of connections that were analyzed, leaving just beta coherence at the STR → STN connection. These findings support the hypothesis that dopamine cell loss acts to increase the sensitivity of the STR to cortical inflow, disrupting the striatum’s role in gating activity to the remainder of the circuit (Magill et al. 2001; Sharott et al. 2017; Tseng et al. 2001).

Notably, our data do not support the hypothesis of beta generation via an autonomous STN/GPe pacemaker network, because directional coherence between the two is heavily attenuated by conditioning with LFPs recorded upstream in the STR and there is significant asymmetry in the NPD with the globus pallidus drive predominating. In agreement, Moran et al. (2011) found evidence for a weakening of the STN-to-GPe feedback connection in the dopamine-depleted state, conflicting with the STN/GPe resonance hypothesis. It may be the case that tight coupling of the STN and GPe results in a near fixed phase relationship in which there is reciprocal coupling, yet from the perspective of phase, the GPe appears to lead.

Estimates of effective connectivity from DCM studies have also suggested that input from cortex to STN is strengthened in the parkinsonian state (Moran et al. 2011), a finding consistent with the idea that dopamine enforces cortical influence on the STN/GPe network (Holgado et al. 2010; Leblois et al. 2006; Magill et al. 2001). It is possible that in PD, cortical activity subsumes the STR as the primary driver of the STN/GPe subcircuit, effectively acting to short-circuit the system. It has been demonstrated that movement is associated with a decreased cortico-pallidal coherence during movement in humans (van Wijk et al. 2017), suggesting that disengagement of cortical influence via this pathway is prokinetic. Thus pathological resonance may arise following dopamine depletion through a compensatory mechanism of increased hyperdirect input following an altered or reduced striatal output (Damodaran et al. 2015; Kumar et al. 2011). In the healthy system it has been proposed that this works to actively decorrelate spiking activity between the two structures (Wilson 2013). The action of dopamine on these inputs is likely to lead to the promotion of beta-amplifying phase alignments between STN and GPe such as that observed by Cagnan et al. (2015).

#### Dopamine depletion is associated with an increased subthalamo-striatal feedback.

Taken together, the analyses presented speak to the existence of a high-beta/low-gamma rhythm that is in general reduced by dopamine depletion. Specifically, our results indicate that connectivity in the frequency band 27–34 Hz is attenuated by the 6-OHDA lesion. Experiments investigating LFPs in the motor cortex of moving rats have demonstrated an increase in activity in this band during movement, suggesting that activity at these frequencies in M2 and SNr is prokinetic (Brazhnik et al. 2012). Our data suggest that high-beta/gamma activity in the normal network is predominantly entrained by the cortex as evidenced by the following: *1*) the unconditioned NPD indicates that gamma is prominently in the forward direction leading from cortex to subcortical sources; and *2*) conditioning the NPD on ECoG recorded at M2 acts to diminish the subcortical directional coherence across a wide band for all connections not involving STN. However, evidence provided by Zold et al. (2012) has demonstrated that oscillatory activity >20 Hz in corticostriatal afferents is not effectively transmitted through the striatum, suggesting that the actual mechanism is likely to be more complicated.

Furthermore, following partialization, some interactions involving STN do remain. In particular, we provide evidence for a significant strengthening of feedback from STN to STR in the lesioned animals in the high-beta/gamma band. We speculate that this signal is facilitated through the strengthening of subcortical loops such as that of the thalamo-striatal pathways (McHaffie et al. 2005). Thalamic afferents make up to at least 25% of input onto spiny projection neurons in the STR (Doig et al. 2010; Smith et al. 2014) but have been far less studied than cortical inputs. Work investigating synaptic remodeling following 6-OHDA depletion in mice has suggested that thalamo-striatal inputs to medium spiny neurons are shifted in favor of the indirect pathway (Parker et al. 2016), perhaps enhancing striatal return of subthalamic activity in a mechanism independent of cortex.

### Segregation of Low-Beta and High-Beta/Gamma Functional Networks

Our analyses suggest a clear separation in the patterns of inter-areal synchronization between low-beta and high-beta/low-gamma frequencies. We find pathological low-beta correlations to be present across large parts of the network and resistant to conditioning with signals from connected structures. In contrast, high-beta/gamma shows a much more hierarchical organization, descending the indirect pathway and possibly looping back subcortically through subthalamic-striatal feedback. Furthermore, high-beta/gamma correlations appear to be weakened by the 6-OHDA lesion.

Multiple studies investigating the electrophysiology of patients with PD (López-Azcárate et al. 2010; Priori et al. 2004) have found evidence for the functional differentiation between low- and high-beta-frequency activity. Low-beta activity is found to be increased by dopamine depletion and correlates with bradykinetic/rigid symptoms in patients, whereas high-beta activity is less responsive to dopamine changes. Interestingly, dopamine replacement in patients has been shown to decouple high- and low-beta frequencies when analyzed with spectral bicoherence (Marceglia et al. 2006). Cortico-subthalamic coherence is also found at this frequency in patients, although again this is largely unresponsive to dopamine (Litvak et al. 2011). We also find evidence for high-beta coherence between BG and cortex, although unlike that found in patients, we find this connectivity to be weakened and shifted to low-beta frequencies by 6-OHDA-induced dopamine depletion.

In the current study we have not made analysis of the interaction or coexistence of the two frequency bands described. This is an interesting problem because the synchronized activity of the subcortical-cortical networks in the beta band is more responsive to dopamine depletion than the synchrony found at the high-beta/gamma frequencies. Future work may utilize tools such as analysis of cross-frequency coupling and time resolved spectral analysis to evaluate the interaction or coexistence of activity in these bands.

### Study Limitations

#### Incomplete signals for conditioning.

The use of partial coherence for inferring neural connectivity is not in itself a novel approach (Eichler et al. 2003; Medkour et al. 2009; Rosenberg et al. 1998; Salvador et al. 2005), and the application of the partialized NPD to LFPs recorded in the rat hippocampus has been previously reported (Halliday et al. 2016). However, these analyses assume that the signals used for conditioning completely capture the activity going through the proposed pathway. However, this is unlikely to be entirely the case, however, because of the finite sampling of the structures afforded from the use of electrodes. That said, the large number of channels used for recordings in the present study ensured that multiple samples were obtained from within each brain structure. In the data presented in this article, subcortical structures were recorded from between two and eight different channels that were all used to condition the estimate of directed coherence. It should also be noted that this sampling limitation is likely to apply most to the larger structures that were analyzed, namely, the motor cortex and striatum, whereas recordings from the smaller sized STN are more likely to capture a larger share of the total activity. This factor must be considered when interpreting conditioning of the NPD with respect to STR signals. It could be the case that M2 → STN connectivity remains in the face of conditioning with the STR LFP as a result of incomplete sampling of neural fields within striatum.

#### Inference of connectivity from nonspiking brain activity.

This study is based on an analysis of mesoscale recordings of brain activity as measured in either the ECoG or the LFP. Transmission of information in the brain is due to axonal propagation of action potentials, and this activity is not explicitly captured by the recording of these signals. LFPs and ECoG comprise a conglomerate of sub- and suprathreshold events that may or may not be tied to spiking activity, and so direct inference of neurophysiological connectivity per se is limited by this. Nonetheless, spike timing has been shown to tightly correlate with negative deflection of the LFP (Destexhe et al. 1999), and increasing evidence that the field itself modulates neural activity is emerging (Goldwyn and Rinzel 2016; Qiu et al. 2015). With respect to the BG, it was previously demonstrated by Mallet et al. (2008a, 2008b) that beta-band activity in the LFPs recorded at STN and GPe of lesioned rats are associated with increased beta-frequency synchronization of action potential firing by neurons in these structures, but see also the report by Magill et al. (2004) that coupling of GPe units and slow-wave activity in the LFP is relatively weak in dopamine-intact rats. Furthermore, we provide evidence for the existence of temporally lagged correlations between rhythmic LFPs recorded between distinct regions of the cortico-BG network that imply causation from one signal to another, a phenomenon that would itself not be possible without the transmission of action potentials. Future work will require an investigation to determine whether directional interactions are ascertainable from multiunit activity and how this relates to lagged synchronization of LFPs.

#### Limits to inference of causal interactions and mechanisms from neurophysiological signals alone.

In this work, we aim to infer how neural activity propagates across the BG network by investigating the statistical relationships between brain signals. The challenges that this approach faces are well documented (Bastos and Schoffelen 2016; Friston 2011). With respect to this study, the benefits that we claim for using a model-free, nonparametric approach (namely, agnosticism to the underlying generating mechanisms of the data) may in turn limit the extent to which inferences can be made. Estimates of directed functional connectivity in this article follow from the assumptions that temporal precedence is indicative of causation. It is however well documented that zero-lag synchronization can emerge from neural circuits with particular (but not unusual) network motifs (Gollo et al. 2014; Vicente et al. 2008; Viriyopase et al. 2012). Additionally, “anticipatory” synchronization, in which positive lags arise from a directed input, has also been described in theoretical neural dynamics (Ambika and Amritkar 2009; Ghosh and Chowdhury 2010; Matias et al. 2011). The anatomically tightly coupled STN-GPe subcircuit is a prime candidate from which these phenomena may permit vanishingly small phase lags that may make the interactions blind to NPD. Answers to these problems may be given in the future by the fitting of biophysical models to the data presented in this article. This would provide a well-defined, quantitative description of the potential mechanisms that act to generate the phenomena we have described.

Furthermore, this study makes inference from the sample statistics of the experimental groups and does not systematically investigate the existence of heterogeneity in the functional connectivity of the group. Such work would likely involve cluster analysis of the connectivity to ask the interesting question of whether localized dopamine depletion can result in a range of distinct individual patterns of beta/gamma propagation.

Finally, we must stress that analysis of functional connectivity cannot access directly the mechanisms that generate sustained neural oscillations and their synchronization. This requires direct experimental manipulations of connections in the network such as that by Tachibana et al. (2011). Nonetheless, the biophysical transmission of rhythmic neural activity and the changes that occur to it following a manipulation such as the 6-OHDA-induced ablation of dopamine neurons leave behind a signature that is accessible to the tools of functional connectivity. Furthermore, the ability to apply systematic “functional lesions” such as that afforded by the conditioned NPD analysis further enables us to infer the generative mechanisms of the observed data.

### Conclusion

Overall, we provide a systematic deconstruction of the propagation of pathological rhythms across the Parkinsonian cortico-basal ganglia circuit in vivo. These findings strengthen our understanding of how normal and pathological rhythms propagate across the network. Our work highlights the importance of considering noncanonical connections in the network, in particular the activity of recurrent subcortical projections that may act to amplify pathological activity within the BG. Future work will aim to understand the exact changes to the network required to generate the patterns of functional connectivity presented in this article, as well as to investigate the relationship with spiking activity in the network.

## GRANTS

This work was supported by Medical Research Council UK Awards UU138197109, MC_UU_12020/5, and MC_UU_12024/2 (to P. J. Magill) and MC_UU_21024/1 (to A. Sharott); Parkinson’s UK Grant G-0806 (to P. J. Magill); and Engineering Research Council UK Awards EPSRC EP/F500351/1 (to T. O. West) and EP/N007050/1 (to D. M. Halliday). S. F. Farmer receives funding from University College London Hospitals Biomedical Research Centre. The Wellcome Trust Centre for Neuroimaging is funded by core funding from the Wellcome Trust (539208).

## DISCLOSURES

No conflicts of interest, financial or otherwise, are declared by the authors.

## AUTHOR CONTRIBUTIONS

T.O.W., L.B., D.M.H., V.L., A.S., P.J.M., and S.F.F. conceived and designed research; A.S. and P.J.M. performed experiments; T.O.W. and D.M.H. analyzed data; T.O.W., L.B., V.L., and S.F.F. interpreted results of experiments; T.O.W. prepared figures; T.O.W., V.L., and S.F.F. drafted manuscript; T.O.W., L.B., D.M.H., V.L., A.S., P.J.M., and S.F.F. edited and revised manuscript; T.O.W., L.B., D.M.H., V.L., A.S., P.J.M., and S.F.F. approved final version of manuscript.
